# Supplementary material for: Saccharomyces cerevisiae does not undergo a quorum sensing-dependent switch of budding pattern
Source: Sci Rep. 2022 May 24;12:8738. doi: 10.1038/s41598-022-12308-z (PMC9130263; doi:10.1038/s41598-022-12308-z)
Supplement: Supplementary file 1 — Supplementary Information 1. [file 41598_2022_12308_MOESM1_ESM.docx]

**FIG S1** Representative ^1^H-NMR spectra of samples. The major resonances used for quantification have been assigned: 1. TSP; 2. Pantothenate; 3. 2-hydroxyisovalerate; 4. Isopropanol; 5. Ethanol; 6. Lactate; 7. Isoamylol; 8. 1-Propanol; 9. Acetate; 10. Hydroxyacetone; 11. Acetoin; 12. Diacetyl; 13. Pyruvate; 14. Succinate; 15. Pyridoxine; 16. Malate; 17. 2-Phenylethanol; 18. 2-oxoglutarate; 19. Choline; 20. Myoinositol; 21. β-Glucose; 22. Trehalose; 23. α-Glucose; 24. Fumarate; 25. Tyrosol; 26. Xanthine; 27. 3-Methylxanthine; 28. Formate; 29. Niacin; 30. Acetaldehyde.

**
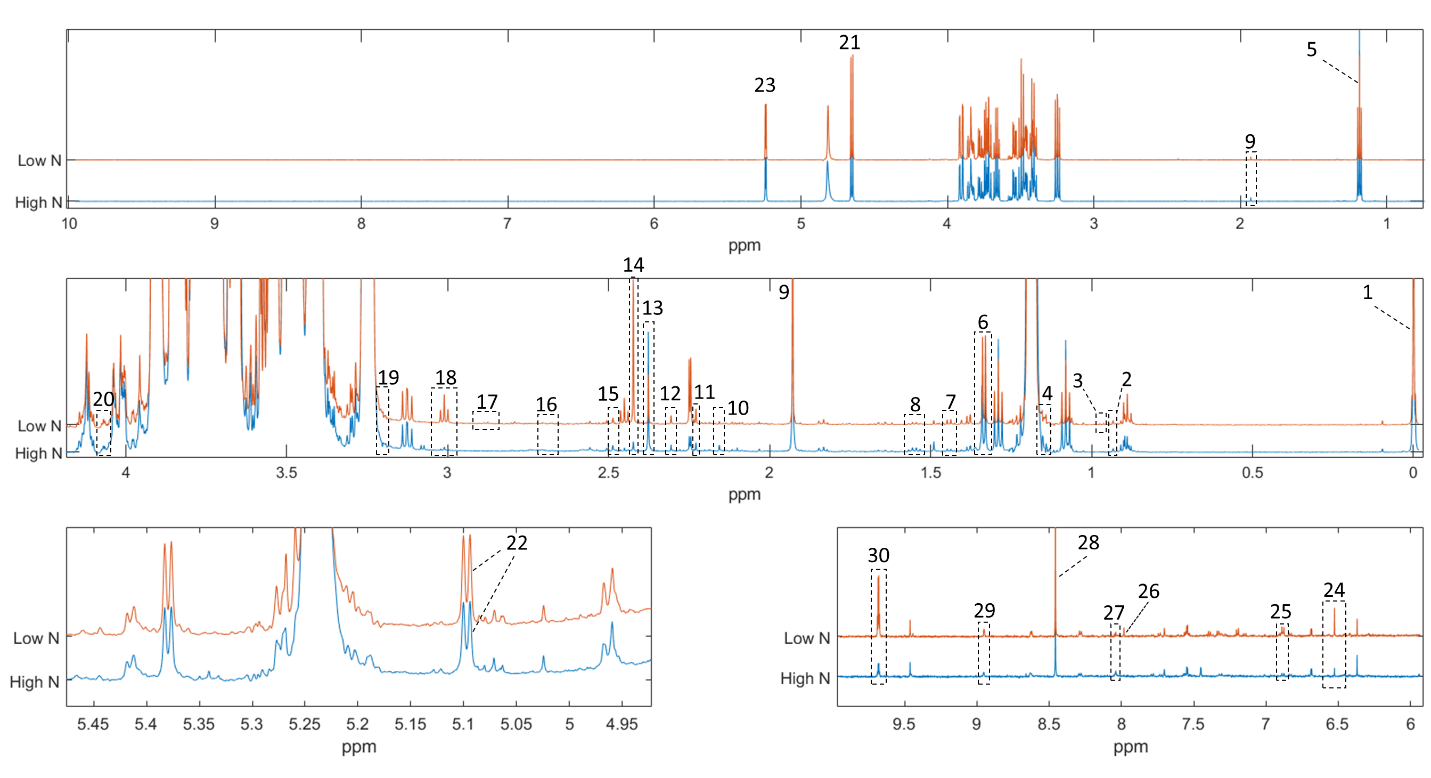
**

**FIG S2** Representative ^1^H-NMR spectra showing the results of the spiking experiment conducted by adding (A) 10 µL 2-phenylethanol and (B) 5 µL tyrosol to the samples.


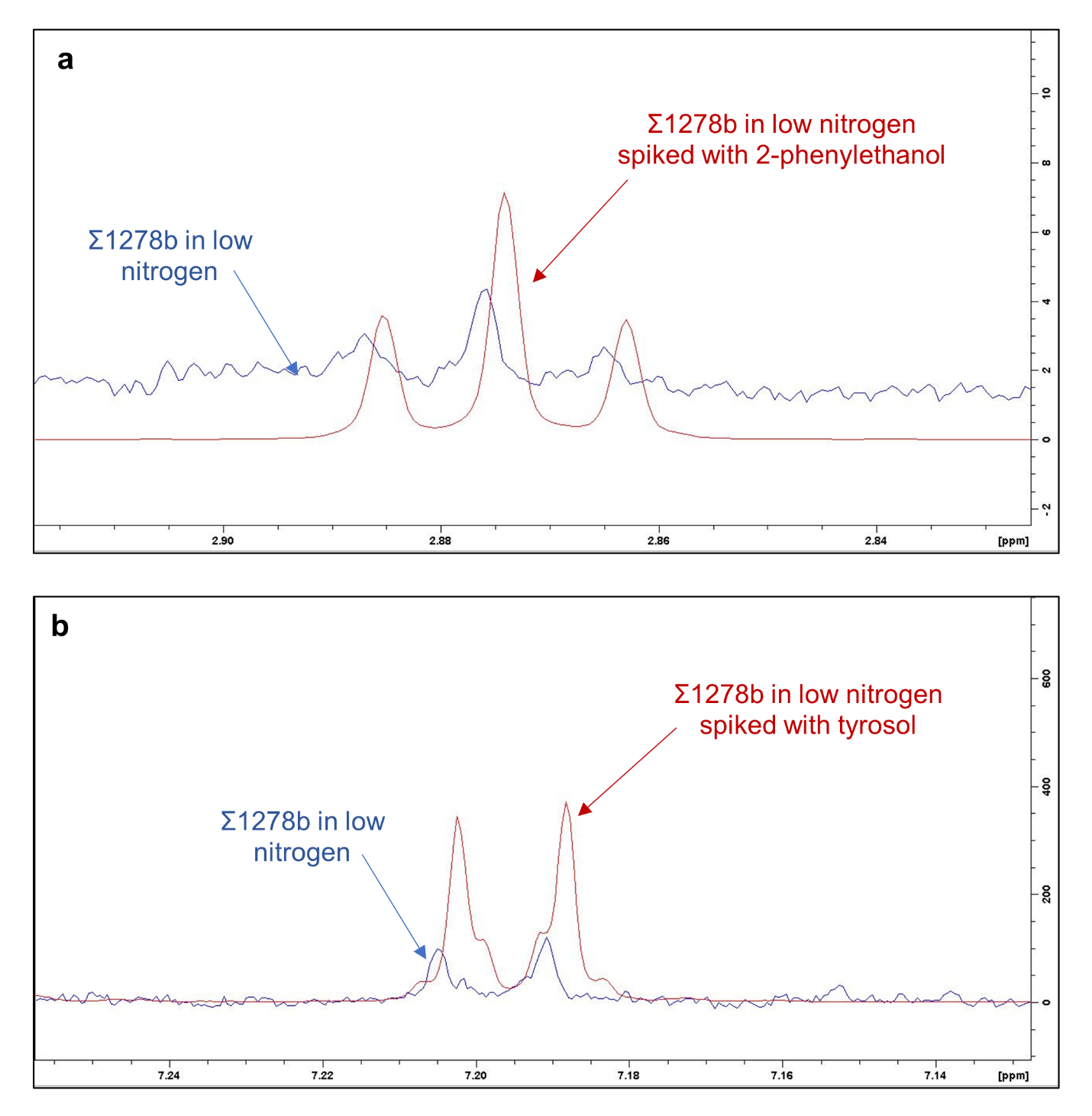


**TABLE S1** Results from Nitrogen Concentration Assay which measured the nitrogen concentration from the media and the supernatant from the three biological replicates A, B and C at the end of the time series experiment i.e. after 30 hours of growth.

| **Nitrogen Condition** | **High Nitrogen** | | | **Low Nitrogen** | | |
| --- | --- | --- | --- | --- | --- | --- |
| **Condition** | **Media** | **Σ1278b** | **S288c** | **Media** | **Σ1278b** | **S288c** |
| **Nitrogen Conc (mM)** | 82.9 | 80.0 | 85.0 | 1.06 | 0.04 | 0.03 |

**TABLE S2** ^1^H-NMR metabolite shifts and intervals with J-coupling. Shifts in bold were used for quantification.

| Metabolite Name | Shift (ppm) | Multiplicity | J-coupling (Hz) | Notes |
| --- | --- | --- | --- | --- |
| 1-propanol | 0.88  **1.53**  3.55 | t  m  t | 7.5  7.1  (6.6) |  |
| 2-hydroxyisovalerate | 0.83  **0.95**  2.01  3.84 | d  d  m  d | 6.86  6.94  (13.81,6.88)  (3.75) |  |
| 2-oxoglutarate | 2.44  **3.01** | t  t | 6.83  6.82 |  |
| 2-phenylethanol | **2.85**  3.83  7.30  7.37 | t  t  m  t | 6.7  -  -  7.5 |  |
| 3-methylxanthine | 3.51  **8.02** | s  s |  |  |
| Acetaldehyde | 2.25  **9.68** | d  q | 3.0  3.0 |  |
| Acetate | **1.92** | s |  |  |
| Acetoin | 1.38  **2.22**  4.41 | d  s  q | 7.1  7.1 |  |
| Arabinose | 3.51  3.68  3.83  3.90  3.95  4.05  4.50  5.25 | dd  m  dd  m  m  m  d  d | (9.7,7.8)  (9.7, 3.5)  7.7  (3.57) | NQ |
| Choline | **3.18**  3.50  4.05 | s  dd  ddd | (5.8,4.1) |  |
| Diacetyl | **2.31** | s |  |  |
| Ethanol | **1.18**  3.66 | t  q | 7.0  7.0 |  |
| Formate | **8.45** | s |  |  |
| Fructose | 3.58  3.69  3.82  3.90  4.00  4.03  4.12 | m  m  m  dd  m  dd  m | (9.9,3.4)  12.8, 1.0 | NQ |
| Fumarate | **6.51** | s |  |  |
| α-glucose | 3.81  3.82  3.38  3.70  3.53  **5.24** | m  m  m  m  m  d | 3.8 | Media nutrient |
| β-glucose | 3.89  3.72  3.38  3.45  3.23  **4.63** | m  m  m  m  dd  d | (7.8, 9.2)  8.0 | Media nutrient |
| Hydroxyacetone | **2.16**  3.27  4.26 | s  s  d |  |  |
| Isoamylol | 0.86  **1.44**  1.66  3.5  4.3 | d  q  m  t  s | 6.9  7.0 |  |
| Isopropanol | **1.16**  4.01 | d  m | 6.0 |  |
| Lactic acid | **1.31**  4.10 | d  q | 7.0  7.0 |  |
| Malic acid | 2.34  **2.66**  4.29 | dd  dd  dd | (15.37,10.2)  15.4, 2.9  (10.23,2.9) |  |
| Myo-inositol | 3.26  3.52  3.61  **4.06** | t  dd  t  t | (9.3)  9.7  2.8 | Media nutrient |
| Niacin | 7.53  8.26  8.61  **8.94** | t  d  d  s | (7.94, 4.90)  (4.98, 1.67) | Media nutrient |
| Pantothenate | 0.88  **0.92**  2.40  3.37  3.4  3.48  3.51  3.98 | s  s  t  s  m  s  s  s |  | Media nutrient |
| Pyridoxine | **2.47**  4.73  7.69 | s  s  s |  | Media nutrient |
| Pyruvate | **2.38** | s |  |  |
| Succinate | **2.41** | s |  |  |
| Taurine | 3.25  3.42 | t  t | 6.1  (6.1) | NQ |
| Trehalose | 3.42  3.64  3.75  3.82  **5.11** | t  dd  m  m  d | (9.47)  (9.93,3.8)  3.8 |  |
| Tyrosol | 2.77  3.77  6.86  **7.17** | t  t  d  d | 6.7  -  8.4  8.5 |  |
